# The effects of hyperbaric oxygen therapy upon ototoxic injuries produced by amikacin in guinea pigs

**DOI:** 10.5935/1808-8694.20130060

**Published:** 2015-10-04

**Authors:** Luciana de Albuquerque Salviano Amora, Adriana de Andrade Batista Murashima, Maria Rossato, Márcia Bento Moreira, Miguel Ângelo Hyppolito, Djalma José Fagundes

**Affiliations:** a MSc (Speech and Hearing Therapist).; b Bachelor degree holder (Technician in the Department of Ophthalmology, Otorhinolaryngology and Head and Neck Surgery at the Ribeirão Preto Medical School - FMRP of the University of São Paulo - USP).; c High school degree holder (Technician in the Department of Ophthalmology, Otorhinolaryngology and Head and Neck Surgery at the Ribeirão Preto Medical School - FMRP of the University of São Paulo - USP).; d PhD (Adjunct Professor II in the Veterinary Medicine Collegiate at the Federal University of the São Francisco Valley - UNIVASF).; e PhD (Professor in the Department of Ophthalmology, Otorhinolaryngology and Head and Neck Surgery at the Ribeirão Preto Medical School - FMRP of the University of São Paulo - USP).; f Associate Professor (Associate Professor in the Department of Surgery at the Federal University of São Paulo - UNIFESP).

**Keywords:** amikacin, cochlea, guinea pigs, hyperbaric oxygenation

## Abstract

Hyperbaric oxygen therapy (HBOT) has enhanced the prevention and treatment of auditory ailments such as ototoxicity.

**Objective:**

To study the effects of HBOT upon ototoxic injuries produced by amikacin.

**Method:**

This experimental study included 12 albino guinea pigs, whose auditory function was assessed through distortion product otoacoustic emissions (DPOAEs) and brainstem auditory evoked potentials (BAEPs) before and after the administration of amikacin (600 mg/kg/day) and HBOT sessions (2 ATA, 60 minutes). Morphological features were analyzed through scanning electron microscopy. Subjects were divided into four groups, as follows: group 1 - saline solution + HBOT; group 2 - amikacin for 8 days; group 3 - amikacin + seven days of rest; and group 4 - amikacin + HBOT.

**Results:**

Group 1 subjects had preserved function and morphology throughout the experiment; group 2 subjects had statistically significant levels of hair cell injury and functional impairment; subjects on groups 3 and 4 had statistically significant functional and morphological impairment after the administration of amikacin, which were still present after the proposed procedures had been carried out.

**Conclusion:**

Hyperbaric oxygen therapy did not change the cochlear hair cell morphology or the electro-physiological thresholds of the guinea pigs given amikacin.

## INTRODUCTION

A wide range of drugs may produce transient or permanent auditory and vestibular impairment as side effects, in what is referred to as drug-related ototoxicity^1^. Aminoglycosides are included in this group of drugs, and are commonly used in the treatment of infectious diseases because of their effectiveness and affordability. Among them, amikacin has an estimated rate of ototoxic events of 13.9%^2^.

Amikacin is used more specifically in pediatric patients^3^, ^4^ with therapeutic and prophylactic purposes, and is broadly prescribed to treat sepsis, meningitis, bacteremia^5^, urinary^6^ and respiratory^7^, ^8^ tract infections. Amikacin has been correlated with decreased perinatal mortality and increased survival in pediatric patients. However, patients are at a significantly higher risk for side effects such as hearing loss^4^.

Experimental studies have shown that intramuscular amikacin at a dosage of 400 mg/kg/day obliterates outer hair cells (OHC) and partially injures inner hair cells (IHC)^9^, ^10^.

On the other hand, hyperbaric oxygen therapy (HBOT), in which individuals are placed in a hyperbaric chamber breathing 100% oxygen at pressures higher than normal atmospheric pressure (between two and three atmosphere absolutes - ATA)^11^, has shown favorable effects for various diseases. Research in the areas of otorhinolaryngology and audiology have resorted to hyperbaric medicine, particularly HBOT, as a means to prevent and treat conditions such as sudden deafness^12^, ^13^, ^14^, noise-induced hearing loss^15^, and ototoxicity^16^.

This study aimed to contribute to the understanding of the inner ear pathophysiological protection mechanisms acting against drug-induced ototoxicity and more specifically the impact of HBOT upon ototoxic injuries introduced by amikacin.

## METHOD

This experimental clinical trial was approved by the Ethics Committee and was granted permits 035/2011 and 407/10. The animals included in the study were male albino guinea pigs (*Cavia porcellus*) of the English strain, weighing approximately 400 grams, selected based on Preyer's reflex.

### The sample

The study included 24 cochleas from 12 guinea pigs. The subjects underwent examination for distortion product otoacoustic emissions (DPOAE) and brainstem auditory evoked potentials (BAEP) under anesthesia with intramuscular ketamine hydrochloride (40 mg/kg) and xylazine (10 mg/kg). Included subjects had to have DPOAEs and BAEPs. Guinea pigs with otitis, ear wax, and narrow ear canals that prevented the placement of an auditory catheter were excluded. The subjects were divided into four groups:

• Group 1: Three guinea pigs (six cochleas) were given subcutaneous saline solution for eight days. Forty-eight hours after the last injection they were submitted to three sessions of HBOT (two ATA for 60 minutes) on days 11, 13, and 15 of the experiment.
• Group 2: Three guinea pigs (six cochleas) were administered subcutaneous amikacin (600 mg/kg/day) for eight consecutive days.
• Group 3: Three guinea pigs (six cochleas) were administered subcutaneous amikacin (600 mg/kg/day) for eight consecutive days. They rested for another seven days and were given subcutaneous saline solution.
• Group 4: Three guinea pigs (six cochleas) were administered subcutaneous amikacin (600 mg/kg/day) for eight consecutive days. Forty-eight hours after the last injection they were submitted to three sessions of HBOT (two ATA for 60 minutes) on days 11, 13, and 15 of the experiment.

### Auditory function assessment

Auditory function assessment was based on DPOAEs and BAEPs. The devices used were the SMART DPOEA^®^ and the SMART EP^®^ from Intelligent Hearing Systems - Miami/Florida (USA).

Auditory function tests were carried out in the beginning of the experiment (in all groups), before HBOT sessions (groups one and four), before resting (group three), and immediately before the subjects were slaughtered (in all groups).

DPOAE tests followed the list of frequencies 2F(1) -F(2) at a ratio F(1)/F(2) = 1.22. Otoacoustic emissions starting at 1.5 kHz were considered. The size of the ear canals of the guinea pigs prevented emissions under this frequency from being assessed, as responses could be mistaken for noise^10^. As described by other authors, the intensity used in this study was 70 dB NPS for F(1) and F(2)^2^, ^16^, ^17^.

The hair in the vertex area and in the region between the orbits was shaved to aid in BAEP capture. The posterior portion of the pinna was cleaned with a steel sponge to remove oil and improve the contact between the electrode and the skin^18^. Surface electrodes were placed as follows: one positive electrode on the cranial vertex, two negative electrodes on the posterior portion of the pinna, and the ground wire on the forehead of the subjects, between their orbits. A layer of electrolytic paste was placed between the tip of the electrodes and the skin of the subjects to improve the conduction of the electric signals^18^.

Insertion earbuds were used to convey the sound stimuli. Alternated clicks of 0.1 ms were played at a rate of 27.7 stimuli per second. Fundamental frequency was 1,000 Hz at an intensity of 90 dB nHL. The signal captured by the electrodes was submitted to high pass and low pass filters of 150 Hz and 3,000 Hz respectively. Filtered amplified signals had their mean values calculated based on 1024 scans of BAEP records with a 12 ms window. The result was provided in the form of electric potential waves^18^. Only wave I was considered in this study, as it recorded the potential of the auditory nerve and is part of the peripheral auditory system^18^. Electrophysiological thresholds were calculated as sound stimuli were played in decreasing steps of 10 dB down to the approximated threshold.

### Hyperbaric oxygen therapy procedure

HBOT sessions were carried in the Department of Experimental Surgery in a small-size multiple-subject experimental hyperbaric chamber. Three sessions were held in alternating days^15^. Each session lasted 100 minutes at 2 ATA, sixty minutes of which at absolute pressure^15^.

### Preparation for scanning electron microscopy (SEM)

The guinea pigs were anesthetized as per the protocol and slaughtered at the end of the observation period by decapitation^19^.

The bulla tympani was opened and 2.5% glutaraldehyde slowly injected through an opening made in the apex of the cochlea and the round window; the specimens were immersed in this solution for four hours at 4°C^9^, ^15^, ^17^, ^18^, ^19^, ^20^. After fixation in glutaraldehyde, the specimens were washed five times^18^ in 0.1 M phosphate buffer solution and dissected to expose the cochlear turns^9^, ^15^, ^17^, ^18^, ^19^, ^20^. After washing, the cochleas were fixated once again in 1% osmium tetroxide and 0.1 M phosphate buffer solution for two hours at 4°C, and washed in 0.1 M phosphate buffer solution at a pH of 7.3^9^, ^15^, ^17^, ^18^, ^19^, ^20^. The specimens were dipped in ethanol in growing concentrations of 50%, 70%, 90%, and 95% for 10 minutes each to be dehydrated. Then they were dipped in 100% ethanol in three cycles of 20 minutes each, and in one last cycle of 12 hours in ambient temperature^9^, ^15^, ^17^, ^18^, ^19^, ^20^. The water still present in the specimens after dehydration was removed through the carbon dioxide critical point drying method. A BAL-TEC CPD 030^®^ (*Critical Point Dryer*) was used in the drying process. The ethanol in the specimens was removed by dipping them in carbon dioxide at 4°C; then the specimens were heated to 40°C to remove the carbon dioxide by evaporation, which happened at 31°C - the critical point^9^, ^15^, ^17^, ^18^, ^19^, ^20^.

The dry specimens were mounted and glued to cylindrical metal stubs using carbon conduction paste^9^, ^17^. In order to allow proper analysis and observation of structures on SEM, the cochleas were covered by a thin layer of gold using a BAL-TEC SCD 050 device. After preparation, the cochleas were electrically conductive for SEM^9^, ^16^, ^17^.

### Criteria for SEM

SEM images were analyzed for OHC counts in the basal, T2 and T3 turns of the cochlea and for presence, absence, or partial presence of inner hair cells in all turns of the cochlea and of outer hair cells in the apical turn of the cochlea.

### Statistical analysis

The Friedman and the Wilcoxon tests were used to assess the differences between DPOAEs. Both tests were used because the study entailed random variables from the same subjects that changed with time. The tests were performed considering a significance level of 95%. The Friedman test was used to assess BAEPs with a significance level of 95%. Each cochlea represented a different, independent experiment, which reflected the temporal evolution of each cochlea. Morphological data was treated using the Kruskal-Wallis test with a significance level of 95%. This analysis considered each different turn of the cochlea, namely the basal, T2, T3, and apical turns, and analyzed them separately. The effects of amikacin and the offered procedures (HBOT and rest) were considered in each case.

Statistical analysis was performed using Microsoft Excel^®^ and software package EXStat^®^ for non-parametric tests^21^.

## RESULTS

DPOAE assessment showed absence of responses in all groups given amikacin as they were tested after eight consecutive days of drug administration. The Wilcoxon test revealed a statistically significant difference between mean signal-to-noise (SNR) ratios before and after amikacin administration (*p* ≤ 0.01).

The DPOAEs of the guinea pigs in groups three and four were absent after eight days of amikacin administration and were still absent and with reduced SNR in both groups after they were submitted to the proposed procedures. Significant differences (*p* ≤ 0.05) were observed in mean SNR after amikacin and after rest or HBOT.

Group 1 had present DPOAEs before and after HBOT. The Wilcoxon test (*p* ≥ 0.05) indicated absence of a significant difference in the subjects’ DPOAEs.

In BAEP testing, the electrophysiological thresholds were increased in groups 2, 3, and 4 after the administration of amikacin and after HBOT or rest. The Friedman test (*p* ≤ 0.05) showed that pre and post-amikacin administration records were statistically different. After amikacin administration, all guinea pigs had electrophysiological thresholds above 90 dB nHL and significant hearing loss.

The study's results showed that the subjects had preserved peripheral auditory systems before the experiment and that these structures were altered and they had hearing loss after the administration of amikacin. Hearing loss was still present after the end of the experiment, regardless of the procedure they were offered after amikacin administration (rest or HBOT).

Group 1 subjects had unaltered thresholds. The Friedman test yielded a *p*-value = 1, showing that the samples were identical in terms of BAEPs.

**Figure 1:**
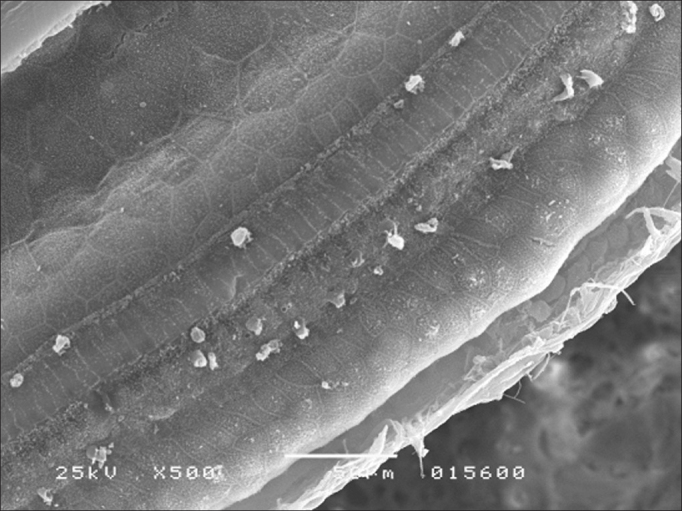
Basal turn of the cochlea of a guinea pig in group 2 showing OHC involvement in all rows and IHC injury (SEM - 1,000x).

Morphological assessment through SEM revealed significant injuries to OHC and IHC in all turns of the cochlea (Figure 1). Inner hair cells of the basal turn were less affected in the groups given amikacin.

**Figure 2:**
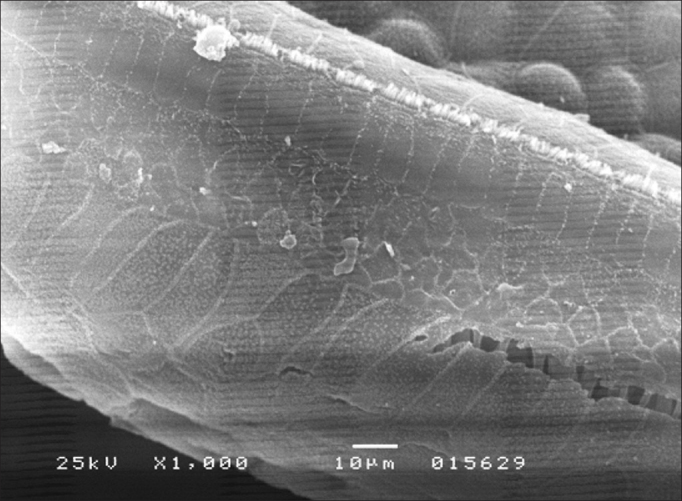
Basal turn of the cochlea of a guinea pig in group 4 showing OHC involvement in all rows and IHC injury (SEM - 1,000x).

**Figure 3:**
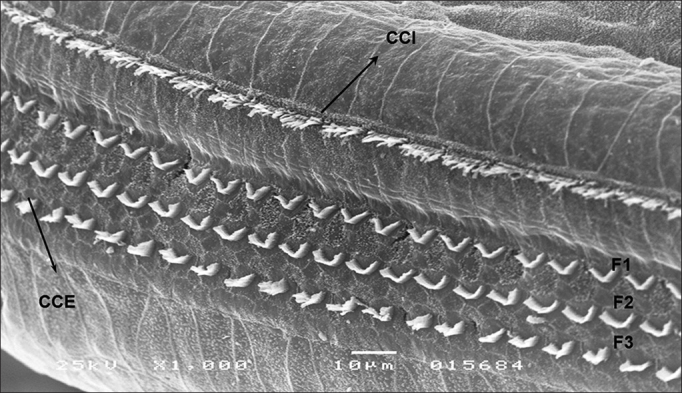
T2 of the cochlea of a guinea pig in group 1 with preserved OHC and IHC. IHC: F1: row 1; F2: row 2; F3: row 3 (SEM - 1,000x).

In group 4, OHC and IHC injuries were seen after eight days of amikacin administration and after HBOT, without significant gains in terms of morphology (Figure 2). Similar results were seen in group 3. IHC and OHC morphology was not altered in group 1, and the three rows of outer hair cells and the row of inner hair cells were preserved (Figure 3).

The Kruskal-Wallis test yielded a *p*-value = 0.5 when OHC counts of the basal, T2, T3, and apical turns of the cochlea for guinea pigs given amikacin minus subjects on group 1 were considered. IHC counts of the basal, T2, T3, and apical turns of the cochlea for guinea pigs given amikacin yielded a *p*-value = 0.22. In both cases, the hypothesis of the groups being statistically similar was rejected.

The morphology of outer and inner hair cells were not altered in group 1 subjects, and all hair cell rows were preserved (Figure 3).

## DISCUSSION

Amikacin is broadly used to treat infectious diseases, particularly in pediatric patients, and has been correlated to significant ototoxicity and irreversible auditory injury. Patients affected by ototoxicity are forced to deal with hearing loss and the socioeconomic barriers imposed by it. The significant costs of rehabilitating these individuals and procuring the usually imported hearing aids must be considered from the point of view of public health.

According to the medical literature, aminoglycosides may be detected in the perilymph 60 to 90 minutes and in hair cells three to four months after systemic drug administration^22^. The adoption of measures to abolish or diminish drug deleterious effects is a challenge to be considered.

### Hyperbaric oxygen therapy (HBOT)

HBOT has been effectively used in other contexts to treat sudden deafness^13^ and offer protection against noise-induced auditory injuries^15^ and ototoxicity by cisplatin^16^.

The auditory protection provided by HBOT has not been clearly described. However, one of the mechanisms involved in amikacin ototoxicity may be related to hair cell ischemia and excessive production of oxygen reactive species accompanied by the typically associated injuries.

HBOT reportedly improves oxidative stress by increasing the production of antioxidants and antioxidant enzymes such as glutathione and superoxide dismutase (SOD), in addition to reducing the levels of markers of oxidative stress such as malondialdehyde and consequently promoting auditory protection by eliminating the oxygen reactive species produced by amikacin.

This study looked into the effects of HBOT upon ototoxic injuries introduced by amikacin to further the understanding of the pathophysiological mechanisms related to inner ear protection.

Ototoxicity was analyzed based on two parameters: morphology and function.

### Morphology

Studies on ototoxicity have routinely resorted to SEM to assess hair cell morphology^9^, ^10^, ^15^, ^17^, ^19^, ^23^.

The data gathered in this study suggest that the subcutaneous administration of amikacin introduced morphologic alterations in the cochleas of the included guinea pigs. HBOT did not offer any protection against cochlear damage.

### Function

DPOAEs have been effectively used to assess OHC function^15^, ^16^, ^23^.

The functional findings related to amikacin ototoxicity reported in this study were in agreement with the findings published in other studies^9^, ^10^, ^17^.

Authors studying ultrastructural and functional cochlear alterations in animal models found extensive OHC lesions after systemic administration of amikacin for 12 consecutive days. These findings were supported by this study and corroborate the ototoxic potential of amikacin^10^.

The guinea pigs treated with amikacin and submitted to HBOT did not have DPOAEs after eight days of drug treatment and had reduced SNR at the end of the experiment, revealing a possible deterioration of OHC function. These findings were also seen in the group submitted to rest after amikacin administration.

Authors have suggested that the degree of DPOAE impairment seen in patients on aminoglycosides is directly correlated to the length of treatment^4^, ^24^, ^25^.

Based on the literature, one may assume that such functional deterioration could be the outcome of the cumulative effect of amikacin, as these findings were seen in the rest and HBOT groups.

Wave I thresholds were increased (> 90 dB nHL) in all groups given amikacin and remained altered after HBOT. Similar findings were seen in the rest group. Neither of the procedures produced statistically significant improvements, as supported by the morphologic findings seen in the subjects.

DPOAEs may be present in subjects with hearing loss of up to 50 dB NA^26^. The data gathered in this study, in which DPOAEs were absent, match the BAEP findings and show the devastating effects of the administered dosage of amikacin as reported in the literature on aminoglycoside-induced ototoxicity^4^, ^9^, ^10^, ^17^, ^27^, ^28^, ^29^.

This study failed to show the auditory protective effects of HBOT reported in the literature^16^; in the cited paper, the authors used intraperitoneal injections of cisplatin (8 mg/kg/day) administered for three consecutive days to induce ototoxicity, submitted the included guinea pigs to HBOT, and reported morphologic and functional improvements in the subjects’ outer hair cells. The number of animals used in this study is consistent with the literature^9^, ^10^, ^15^, ^16^ and was based on the recommendations of the Research Ethics Committee; the differences found in our study cannot be attributed to this variable. However, it is possible that differences in metabolic pathways and modes of injury may have had a determining role.

### Closing remarks

The hyperoxia caused by HBOT increases tissue tolerance to ischemia and enhances the biologic response against oxygen reactive species. Future studies with similar methodology should consider different times of exposure to the same dosages of amikacin and the same HBOT protocol described in this study. Oxidative stress improvements secondary to HBOT by increases in the production of antioxidants and antioxidant enzymes such as glutathione and superoxide dismutase could be more thoroughly assessed, as well as reductions in oxidative stress markers such as malondialdehyde.

## CONCLUSION

• HBOT did not lead to significant improvements in the morphology of the hair cells exposed to amikacin.
• The electrophysiological thresholds of the guinea pigs given amikacin were not significantly improved after HBOT, and remained equal to or greater than 90 dB nHL.
• HBOT did not improve the outer hair cell function of the guinea pigs given subcutaneous amikacin.
